# Identification of Dendritic Cell Maturation, TLR, and TREM1 Signaling Pathways in the *Brucella canis* Infected Canine Macrophage Cells, DH82, Through Transcriptomic Analysis

**DOI:** 10.3389/fvets.2021.619759

**Published:** 2021-03-19

**Authors:** Woo Bin Park, Suji Kim, Soojin Shim, Han Sang Yoo

**Affiliations:** ^1^Department of Infectious Diseases, College of Veterinary Medicine, Seoul National University, Seoul, South Korea; ^2^BK21 Four Future Veterinary Medicine Leading Education and Research Center, Seoul National University, Seoul, South Korea; ^3^Research Institute for Veterinary Science, Seoul National University, Seoul, South Korea; ^4^BioMax/N-Bio Institute, Seoul National University, Seoul, South Korea

**Keywords:** *Brucella canis*, RNA-Seq, transcriptomic analysis, TLR signaling, early immune response

## Abstract

Research has been undertaken to understand the host immune response to *Brucella canis* infection because of the importance of the disease in the public health field and the clinical field. However, the previous mechanisms governing this infection have not been elucidated. Therefore, *in vitro* models, which mimic the *in vivo* infection route using a canine epithelial cell line, D17, and a canine macrophage, DH82, were established to determine these mechanisms by performing an analysis of the transcriptomes in the cells. In this study, a coculture model was constructed by using the D17 cell line and DH82 cell line in a transwell plate. Also, a single cell line culture system using DH82 was performed. After the stimulation of the cells in the two different systems infected with *B. canis*, the gene expression in the macrophages of the two different systems was analyzed by using RNA-sequencing (RNA-seq), and a transcriptomic analysis was performed by using the Ingenuity Pathway Analysis (IPA). Gene expression patterns were analyzed in the DH82 cell line at 2, 12, and 24 h after the stimulation with *B. canis*. Changes in the upregulated or downregulated genes showing 2-fold or higher were identified at each time point by comparing with the non-stimulated group. Differentially expressed genes (DEGs) between the two culture models were identified by using the IPA program. Generally, the number of genes expressed in the single cell line culture was higher than the number of genes expressed in the coculture model for all-time points. The expression levels of those genes were higher in the single cell line culture (*p* < 0.05). This analysis indicated that the immune response-related pathways, especially, the dendritic cell maturation, Triggering receptor expression on myeloid cells 1 (TREM1) signaling, and Toll-like receptor (TLR) signaling pathway, were significantly induced in both the culture systems with higher *p*-values and *z*-scores. An increase in the expression level of genes related to the pathways was observed over time. All pathways are commonly associated with a manifestation of pro-inflammatory cytokines and early immune responses. However, the Peroxisome proliferator-activation receptor (PPAR) signaling and Liver X Receptor/Retinoid X Receptor (LXR/RXR) signaling associated with lipid metabolism were reduced. These results indicate that early immune responses might be highly activated in *B. canis* infection. Therefore, these results might suggest clues to reveal the early immune response of the canine to *B. canis* infection, particularly TLR signaling.

## Introduction

Brucellosis is a reemerging worldwide zoonotic disease caused by the genus *Brucella*. *Brucella* spp. such as *Brucella melitensis* and *Brucellar suis*, are the facultative intracellular pathogens that are commonly able to overcome the host innate immunity during the early infection. *Brucella* spp. affecting not only the immune response but also the killing action of macrophages migrate within phagocytic vacuoles and replicate in cell vesicles, causing chronic infection (1). Among these species, *Brucella canis* is known as a cause of canine brucellosis and, similar to the other *Brucella* spp., is also a cause of the zoonotic disease that can occur in humans. The host of *B. canis* is mainly dogs, and granulomatous lesions are identified in various organs during the infection and are characterized by reproductive disorders such as abortion in females and epididymitis and prostatitis in male dogs (2). When an infection occurs in a human, mild and non-specific symptoms usually occur and then antibiotic therapy is usually applied. Infection with *B. canis* can occur through the conjunctival or oronasal route or along the venereal route, and can also occur through contaminated milk, urine, aborted fetuses, vaginal secretion, and semen (3–5). Human infection with *B. canis* is known to occur very rarely, but the frequency has been increasing in recent years. The prevalence has been increasing due to the increase in unsanitary kennel facilities, companion dogs, and stray dogs. *B. canis* is currently reported in many parts of the world, and is considered endemic in the USA, Latin America, and Mexico (6–11). Infections of *B. canis* have also been reported in Asian countries, such as China and Japan, in Africa, and in European countries, such as Germany, the United Kingdom, and Italy (12–18). The prevalence of *B. canis* infection in 2,394 dogs, including the companion and the stray dogs in Korea was examined. The prevalence was found to be significantly higher in dogs older than 6 years and in female dogs (19).

Immunological studies of *B. canis* infection have also been conducted. Usually, research has focused on the expression of cytokines following the infection of various cell lines with *B. canis* (20–23). Previous studies have shown that the immune responses of humans to brucellosis are similar to those of animals to brucellosis (21). In addition, the immune response of human and canine dendritic cells to *B. canis* infection has been compared based on the expression of cytokines (22). Many attempts have been made to understand the immunopathological mechanism of *B. canis* infection because of the importance of the disease in recent public and clinical realms (2, 21). To date, however, the mechanisms governing *B. canis* infection have not been elucidated.

Therefore, in this study, an *in vitro* coculture model was established by using the D17, a canine epithelial cell line, and the DH82, a canine macrophage cell line. The coculture model is designed to observe the host response to the pathogen by interaction between epithelial cells and immune cells in comparison to the single cell line culture model. The coculture model was applied to investigate the host immune response to *B. canis* infection through a transcriptomic analysis of the DH82 cells using total RNA-sequencing (RNA-seq).

## Materials and Methods

### Cell Culture and Stimulation

*Brucella canis* QE13, from the College of Veterinary Medicine of Gyeongsang National University, was cultured on *Brucella* broth (BD, NJ, USA) at 37°C under aerobic conditions. For the stimulation of the D17 cell line and DH82 cell line, the bacteria were cultured up to the exponential growth phase, reaching 3.06E + 09 CFU.

In the coculture model, ~5.0 × 10^5^ cells/well of the D17 cell line (ATCC CCL-183) were seeded onto the apical side of a Transwell insert (Transwell permeable support; Corning, MA, USA) and incubated for 3 h in DMEM (Gibco, NY, USA) containing 15% fetal bovine serum (FBS; Gibco, NY, USA) at 37°C in a humidified chamber containing 5% CO_**2**_. After the D17 cell line was stabilized, 5.0 × 10^5^ cells/well of the DH82 cell line (ATCC CRL-10389) were seeded onto the Transwell plate well with DMEM containing 15% FBS. After the DH82 cells were stabilized, the D17 cell line where the apical side of Transwell insert was stimulated by using Dulbecco's Phosphate-Buffered Saline (DPBS; negative control) and *B. canis* at an multiplicity of infection (MOI) of 10:1.

In the single cell line culture model, 5.0 × 10^5^ cells/well of the DH82 cell line were seeded onto one well of a 12-well plate (Corning, MA, USA) with DMEM containing 15% FBS. After the DH82 cell line was stabilized, it was stimulated by using DPBS (negative control) and *B. canis* at an MOI of 10:1.

Each experiment was conducted three times.

The D17 cell line and DH82 cell line were purchased through ATCC (https://atcc.org/).

### Invasion Assay

The infection of *B. canis* to the DH82 cell line was performed at MOI of 1:10. After the infection, it was incubated for 12 h at 37°C in a humidified chamber containing 5% CO_2_. Each well was thoroughly washed with PBS after the incubation. For the quantification of intracellular bacteria, the infected monolayer was cultured at a place with 100 μg/ml of gentamicin (Sigma, St. Louis, USA) for 2 h to kill extracellular bacteria. After the antibiotics treatment, the cells were washed out with PBS to remove residual antibiotics and lysed with 0.2% Triton-X100 at 2, 12, and 24 h after the removal. Lysates were plated on agar after a serial dilution and measured colony-forming units (CFUs) after 48 h at 37°C in a humidified chamber containing 5% CO_2_. These experiments were triplicated.

### RNA Extraction

After 2, 12, and 24 h of the incubation, total RNA was extracted from the DH82 cell line using the RNeasy Mini Kit (Qiagen, Hilden, Germany) according to the instructions of the manufacturer. RNA purity and integrity were evaluated by determining the OD 260/280 ratio and an analysis using the Agilent 2100 Bioanalyzer (Agilent Technologies, CA, USA). The total RNA concentration was measured by using the Quant-IT Ribogreen (Invitrogen, CA, USA). To determine the integrity of the total RNA, the TapeStation RNA Screen Tape system (Agilent Technologies, CA, USA) was used. Only high-quality RNA preparation, with RNA integrity numbers (RINs) > 7.0, was used for the RNA library construction.

### Transcriptomic Analysis

The libraries were prepared for 100-bp paired-end sequencing using a TruSeq Stranded mRNA Sample Preparation Kit (Illumina, CA, USA). Specifically, mRNA molecules were purified and fragmented from 1 μg of total RNA using oligo (dT) magnetic beads. The fragmented mRNAs were synthesized as a single-stranded complementary DNA (cDNA) using a Random Hexamer Primer (Kapa Biosystems, MA, USA). By applying cDNA as a template for the second strand synthesis, a double-stranded cDNA was prepared. After sequential end repair, A-tailing, and adapter ligation, the cDNA libraries were amplified by using a PCR. The quality of these cDNA libraries was evaluated using the Agilent 2100 Bioanalyzer (Agilent, CA, USA). These values were quantified using the KAPA library quantification kit (Kapa Biosystems, MA, USA) according to the library quantification protocol of the manufacturer. Following the cluster amplification of denatured templates, sequencing was progressed as paired end (2 × 150 bp) by using an Illumina platform sequencer (Illumina, CA, USA). Each gene was compared with the results of 0 h to proceed with the analysis.

#### Filtering

Low-quality reads were filtered according to the following criteria: reads containing more than 10% of the skipped base reads (marked as “N”s'), reads containing more than 40% of the bases whose quality score was < 20, and reads whose average quality scores are per read was < 20. The whole filtering process was performed using the in-house scripts.

#### Sequencing Alignment

The filtered reads were mapped to the reference genome species using the aligner TopHat (24).

#### Gene Expression Estimation

The gene expression level was measured by Cufflinks v2.1.1 (25) using the human gene annotation database. To improve the accuracy of the measurement, the multi-read-correction option and the frag-bias-correct option were applied. All other options were set to default values.

#### Differentially Expressed Gene Analysis

Differentially expressed gene (DEG) analysis was performed by using Cuffdiff (26). To enhance the analysis accuracy, the multi-read-correction option and the frag-bias-correct option were applied. All other options were set to default values. DEGs were identified based on a *q*-value threshold < 0.05 for correcting the errors caused by multiple testing (27).

#### Gene Ontology Analysis

The gene ontology (GO) database classifies the genes according to the three categories, such as biological process (BP), cellular component (CC), and molecular function (MF), and provides information on the functions of genes. To characterize the identified genes from the DEG analysis, a GO-based trend test was carried out using the Fisher's exact test (28).

#### Biological System Analysis

The data were analyzed using IPA (Qiagen, Hilden, Germany, https://www.qiagenbioinformatics.com/products/ingenuityphathway-analysis). DEGs with adjusted values of *p* < 0.05 were obtained by using the IPA program. Each gene was mapped to its corresponding gene gain using the Ingenuity Knowledge Base. Biological function analysis was performed using IPA to compare DEG associated with the disease and function, the molecular and cellular function, and physiological system function in DH82 cells treated with *B. canis* at each time point. The canonical pathway for the *B. canis*-treated DH82 cells was also identified through the canonical pathway of the IPA library (29).

#### Comparison Analysis

The data were analyzed by using IPA (Qiagen Inc., Hilden, Germany, https://www.qiagenbioinformatics.com/products/ingenuityphathway-analysis). Using the data, a comparative analysis was conducted according to each time and each culture model. However, the difference in DEGs with respect to time was confirmed, and the difference in DEGs according to the model type was confirmed.

### Expression Analysis of Selected Genes by Using Quantitative Real-Time PCR

The expression of levels of five genes, IL6, CCL5, CXCL10, CXCL8, and IL1B, in RNA-seq at 2, 12, and 24 time points were compared with those from the quantitative real-time PCR (qRT-PCR) of the two different experiments. The qRT-PCR was performed by using 1 μl of cDNA, a Rotor-Gene SYBR Green PCR Kit (Qiagen Inc., Hilden, Germany), and a Rotor-Gene Q real-time PCR cycler (Qiagen, Hilden, Germany) (Table 1). The cycling condition is as follows: 95°C for 3 min, followed by 45 cycles of 95°C for 15 s, 30 s at 60°C, and 30 s at 72°C with a fluorescence detected during the extension phase. The expression level was determined by the 2^−ΔΔCt^ method using glyceraldehyde-3-phosphate dehydrogenase (GAPDH) as a reference gene. The relative expression levels were compared to the results of the control cells to determine expression log_2_ (fold change) for each gene.

**Table 1:** Canine forward and reverse primers for validation by quantitative real-time PCR.

**Target**	**Forward primer**	**Reverse primer**	**Accession number**
IL-6	5′-CTGGCAGGAGATTCCAAGGAT-3′	5′-TCTGCCAGTGCCTCTTTGC-3′	NM_001003301
CCL5	5′-CAGAAGAAATGGGTGCGGGAGTA-3′	5′-CAAGAAGCAGTAGGAAAGTTTGCATG-3′	NM_001003010
CXCL10	5′-TCCTGCAAGTCCATCGTGTC-3′	5′-ATTGCTTTCACTAAACTCTTGATGGTC-3′	NM_001010949
CXCL8	5′-GACAGTGGCCCACAATGTGAAAACTC-3′	5′-GTTGTTTCACGGATCTTGTTTCTCAGC-3′	NM_001003200
IL1β	5′-GGAAATGTGAAGTGCTGCTGCCAA-3′	5′-GCAGGGCTTCTTCAGCTTCTCCAA-3′	NM_001037971

### Statistical Analysis

Statistical significance of internalization was analyzed by using the Student's *t*-test or repeated measures ANOVA using Graphpad Prism version 7.00 (Graphpad Software, San Diego, CA, USA, https://www.graphpad.com). All genes were considered to be differentially regulated when the value was *p* < 0.05. In the case where the difference was found to be significant, the fold change value was expressed as the control condition and it is mentioned as follows: fold change = the mean ratio of gene expression in the bacteria-treated cells/the mean ratio of gene expression in the DPBS-treated cells.

## Results

### Invasion Assay

The invasibility of the *B. canis* was evaluated *in vitro* using the DH82 cell line. It has been confirmed that intracellular bacteria increase over time (Supplementary Figure 1). These results suggest that the changes of gene expression in the DH82 cell line were caused by *B. canis*, not by other external causes. Also, the results indicate that intracellular *B. canis* might induce immune responses in the host cells through the intracellular survival of the bacterium.

### Differentially Expressed Genes

**Figure 1:**
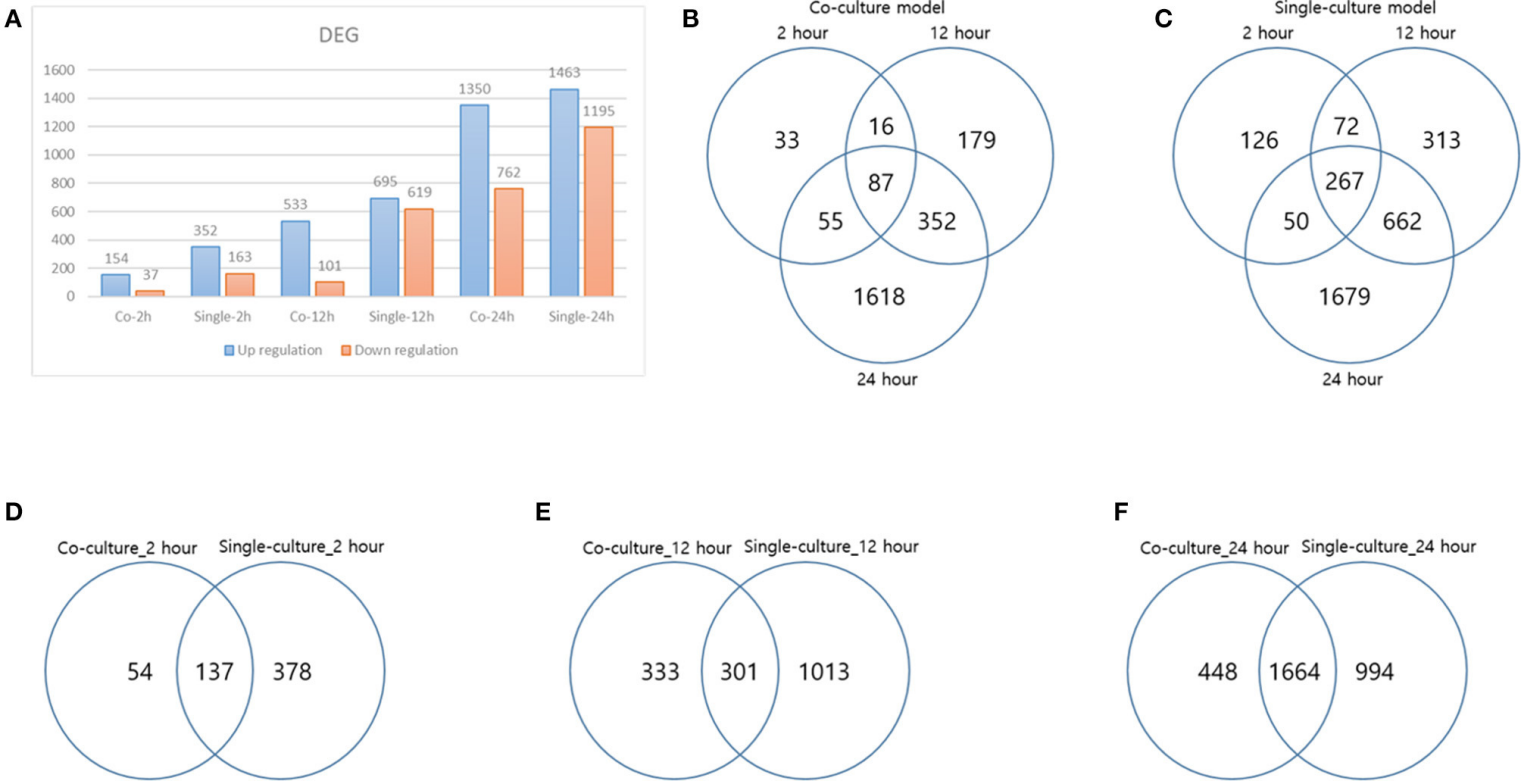
Differentially expressed genes (DEGs) result. **(A)** DEG result over each time of each model, **(B)** DEG distribution over time in a coculture model, **(C)** DEG distribution over time in a single-culture model, **(D)** DEG comparison of each model at 2 h, **(E)** DEG comparison of each model at 12 h, and **(F)** DEG comparison of each model at 24 h.

Each gene was analyzed for a change in the expression through the fold change value compared to 0 h. By stimulating cells with *B. canis*, DEGs with changes higher than 2-fold in the coculture model were 191, 634, and 2,112 at 2, 12, and 24 h, respectively, while DEGs in the single cell line culture model were identified as 515, 1,314, and 2,658 at different time points, respectively. The number of genes with altered expression in the single cell line culture model was higher than the coculture model. The results were compared with the two models in each time period and compared with respect to each model time period. About 87 and 267 DEGs were commonly identified at all-time points in the coculture and single cell line culture model, respectively. In comparison of the two models at the same time point, 137, 301, and 1,664 DEGs were commonly identified in 2, 12, and 24 h, respectively (Figure 1). From 2 or more samples, 30 genes were selected from the highest alternation in gene expression at each time period and each culture model. In five or more samples, 17 DEGs (ACOD1, CCL3L3, CCL4, CCL5, CXCL5, CXCL8, CXCL10, FST, GPR84, IL1A, IL1B, IL6, IL23A, OLR1, PTGS2, RASSF6, and SAA1) were upregulated, three DEGs (CACNA2D1, FHIT, and TSHZ2) were upregulated only in the coculture models, and six DEGs (FCRL2, INHBA, MMP3, MMP10, MMP13, SERPINB2, and SMPDL3A) were upregulated only in the single cell line culture models. Two DEGs (TNFAIP2 and STEAP4) were upregulated at the 2-h results in both the culture models, and three DEGs (SRGN, OSM, and F3) were upregulated at the 24-h results in both the culture models (Table 2). In four or more samples, nine DEGs (ADGRF1, CD180, FGFR2, FMN2, HUNK, MRVI1, SERTAD4, SLC27A6, and TRPM2) were found to be downregulated. The expression of five DEGs (ANKRD66, MLLT11, SORBS2, TMEM273, and WDR31) was downregulated only in the coculture models, and the two DEGs (CAVIN1 and TRIM72) were downregulated in the single cell line culture models. Only one DEG (SIRPB1) showed a downregulated expression only in the 2-h results of both the culture models, and the three DEGs (FZD1, PADI4, and SYPL2) showed a downregulated expression only in the 24-h results of both the culture models (Table 3).

**Table 2:** Differentially expressed genes (DEGs) identified for each model and each time.

**Gene symbol**	**Fold changes in coculture model**			**Fold changes in single-culture model**		
	**2 h**	**12 h**	**24 h**	**2 h**	**12 h**	**24 h**
FHIT	89.264	83.286	86.223	–	–	–
ACOD1	64.445	18.126	53.446	156.498	71.012	–
CXCL10	55.330	26.723	–	168.897	95.010	–
CCL4	45.887	13.642	410.148	149.086	184.823	302.334
OLR1	33.128	28.840	270.597	49.522	36.504	57.282
FFAR2	29.243	9.646	88.035	99.044	–	–
CCL5	22.943	18.636	63.119	63.558	81.572	85.627
SAA1	22.471	28.051	198.088	95.010	436.549	826.001
CCL3L3	20.393	22.316	719.076	60.969	286.026	588.134
IL1A	19.427	13.454	704.277	84.449	337.794	608.874
SAA1	17.388	21.407	116.970	74.028	328.557	481.036
NEURL3	16.679	13.086	54.569	27.284	–	–
IL1B	16.679	13.929	1002.926	77.708	1351.176	1144.102
SAA1	16.223	24.251	116.970	86.223	354.588	398.932
IL6	14.026	12.126	202.251	54.569	421.679	471.136
CXCL8	12.295	–	89.884	34.060	138.141	181.019
GPR84	12.042	10.556	88.035	19.562	52.346	–
FST	10.056	–	–	25.634	43.111	–
CXCL5	8.456	10.483	97.681	16.450	174.853	138.141
TNFAIP2	7.516	–	–	19.293	–	–
STEAP4	7.062	–	–	23.425	–	–
RASSF6	6.409	–	–	18.126	45.887	–
PTGS2	6.320	–	272.479	21.857	95.670	396.177
IL1RN	5.242	–	58.081	17.030	–	–
EGLN3	–	25.813	110.661	−3.182	–	108.383
TSHZ2	–	7.621	56.493	–	–	–
IL23A	–	–	1009.902	29.041	76.639	1418.352
SRGN	–	–	91.139	–	–	56.103
OSM	–	–	88.035	–	–	237.207
F3	–	–	48.840	–	–	75.061
LIF	–	–	48.503	–	38.586	–
INHBA	–	–	–	–	134.364	240.518
MMP3	–	–	–	–	54.569	247.280
MMP13	–	–	–	–	44.324	83.865
MMP10	–	–	–	–	38.854	168.897
FCRL2	–	–	–	–	36.252	64.000
SMPDL3A	–	–	–	–	33.359	53.446
SERPINB2	–	–	–	–	31.341	59.714

*The main DEGs in which upregulation occurred in each experiment*.

**Table 3:** DEGs identified for each model and each time.

**Gene symbol**	**Fold changes in coculture model**			**Fold changes in single-culture model**		
	**2 h**	**12 h**	**24 h**	**2 h**	**12 h**	**24 h**
SIRPB1	−4.823			−10.126		
HTR1D	−2.657	−5.464	−19.293		−27.096	
MLLT11	−2.297	−3.506				
SORBS2	−2.219	−5.856				
TMEM273	−2.189					–
GRIA4	−2.144	−3.605				
ADGRD1		−4.724			−6.727	
ANKRD66		−4.347	−7.062			
MRVI1		−4.141	−9.849			−12.381
WDR31		−4.000	−11.158			
FMN2		−3.580	−9.254			−17.388
LGR6		−3.531		−3.294	−8.340	
FGFR2		−3.506	−18.507			−12.126
DHRS3		−3.387		−3.227	−9.448	
SLC27A6		−3.053	−9.254			−12.817
SERTAD4			−33.359		−7.516	−49.867
ADGRF1			−26.909		−7.516	−170.072
FZD1			−15.562			−15.032
CD180			−13.929		−19.973	−38.055
SMAD6			−12.906		−6.916	
SYPL2			−11.876			−13.929
HUNK			−11.392		−6.964	−16.679
TRPM2			−10.411		−8.515	−24.761
PADI4			−10.267			−12.729
RAB36			−7.210		−7.413	
NIPAL1			−7.013		−10.339	
CAVIN1				−3.160	−6.964	
TRIM72					−7.210	−40.224

*The main DEGs in which downregulation occurred in each experiment*.

Raw files and normalized data sets are available from the Gene Expression Omnibus (GEO) https://www.ncbi.nlm.nih.gov/geo under the accession number GSE134331 (https://www.ncbi.nlm.nih.gov/geo/query/acc.cgi?acc=GSE134331).

### Canonical Pathway Analysis

Of all DEGs that mapped to the Ingenuity Knowledge Base and passed the data set filter (*p*-value), 191, 634, 2,112, 515, 1,314, and 2,658 DEGs of the DH82 cells treated with *B. canis* at 2, 12, and 24 h and each culture model, respectively, were analyzed. All DEGs were used in a core analysis, which was carried out by using Ingenuity® Pathways Analysis (IPA, Ingenuity Systems, https://www.ingenuity.com). Canonical pathways were identified for the DEGs of the DH82 cells treated with *B. canis* at each time point and each culture model. The canonical pathway was analyzed for each time period and each culture model, and was compared with the same time period of different culture models using a comparative analysis according to the passage of time for each model. In most cases, immunity-related pathways were predominantly identified (Table 4).

**Table 4:** Major canonical pathways related to immune response.

**Pathway**	**Coculture model**						**Single cell culture model**					
	**2 h**		**12 h**		**24 h**		**2 h**		**12 h**		**24 h**	
	***p*-value**	***z*-score**	***p*-value**	***z*-score**	***p*-value**	***z*-score**	***p*-value**	***z*-score**	***p*-value**	***z*-score**	***p*-value**	***z*-score**
Communication between adaptive immune cells and innate immune cells	1.10E-15	NaN	4.74E-11	NaN	1.17E-09	NaN	1.98E-11	NaN	3.52E-08	NaN	7.77E-07	NaN
Acute phase responses	5.49E-08	2.714	2.15E-04	3.317	5.77E-06	2.828	2.76E-10	2.683	1.87E-09	2.556	3.69E-04	2.828
Dendritic cell maturation	2.64E-12	3.742	1.61E-03	2.714	1.07E-06	4.564	5.82E-09	4.359	1.59E-07	4.041	1.86E-05	4.333
Toll-like receptor signaling	1.02E-09	1.633	1.56E-06	2.121	3.38E-09	2.668	2.30E-09	2.333	7.04E-08	2.887	1.79E-04	2.887
TREM1 signaling	1.18E-15	3.207	9.47E-08	3.606	3.83E-13	5.112	3.35E-14	3.771	3.42E-09	3.578	8.24E-08	4.315
Role of pattern of recognition receptors of bacteria and viruses	4.03E-10	2.828	2.48E-11	2.887	1.65E-12	3.838	9.08E-09	2.530	3.81E-12	2.500	8.01E-11	3.000
NF-κB signaling	4.44E-10	2.138	1.37E-05	2.500	4.08E-12	2.480	7.09E-09	1.147	3.83E-12	2.000	2.69E-07	3.429
IL-6 signaling	9.64E-10	3.464	2.68E-04	3.317	1.55E-08	4.352	1.72E-12	4.025	1.62E-12	3.772	1.04E-05	4.596
Role of cytokines in mediating communication between immune cells	1.41E-08	NaN	1.47E-05	NaN	8.89E-03	NaN	2.99E-06	NaN	2.05E-04	NaN	1.86E-02	NaN
HMGB1 signaling	9.79E-11	3.464	6.58E-07	3.606	6.45E-09	3.528	2.08E-11	3.638	8.80E-14	3.286	3.20E-06	3.528

*List of canonical pathways organized according to z-score and p-value. Pathway with z-score value NaN means the pathway that has not been identified in the data contained within the IPA program but has been significantly expressed through p-value in the results of this study*.

**Figure 2:**
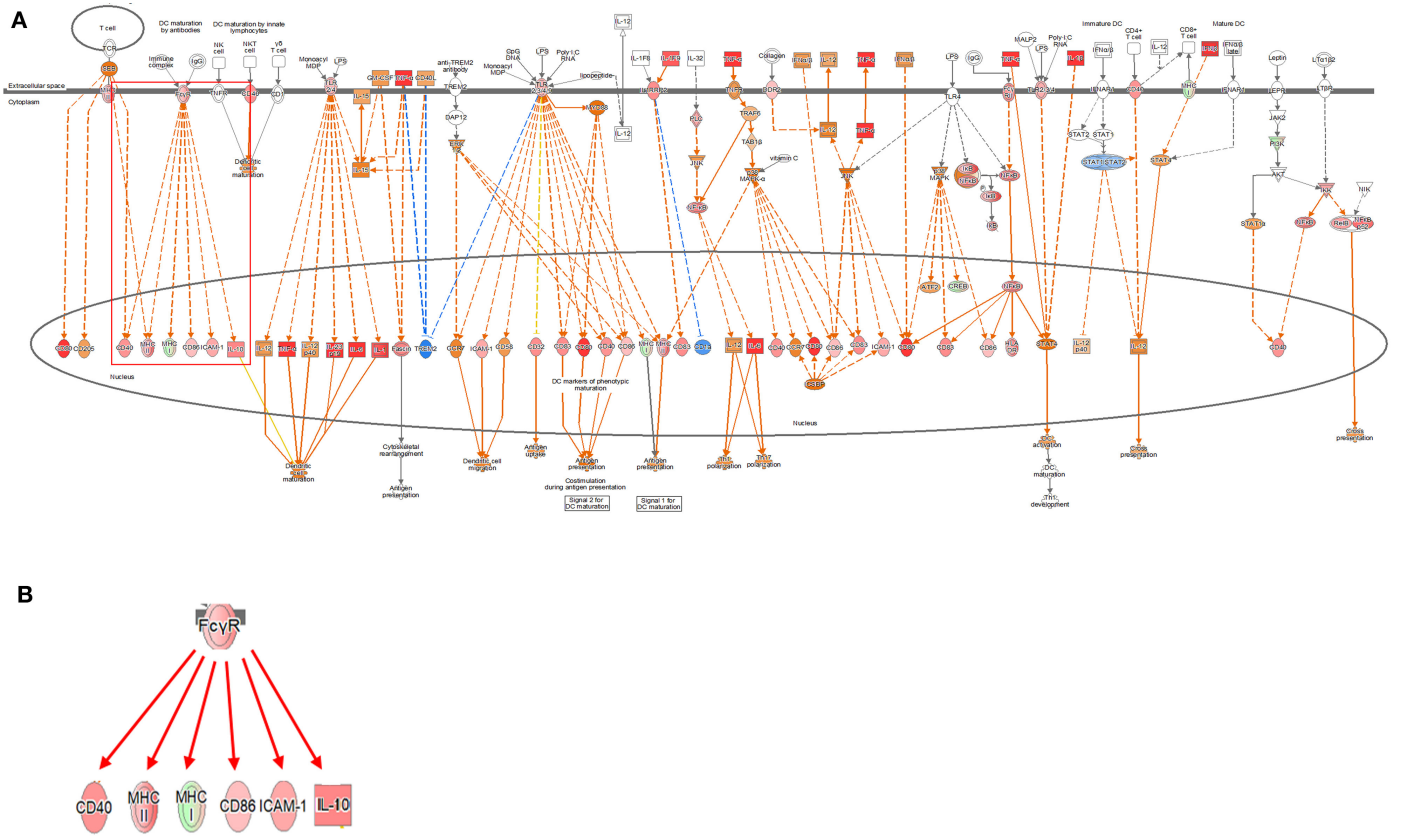
Ingenuity pathway analysis of the dendritic cell maturation signaling pathway in the DH82 cell line treated with *Brucella canis*. **(A)** Dendritic cell maturation pathway in a 24 h on coculture model **(B)** Fc gamma receptor- (FcγR-) associated pathway on dendritic cell maturation pathway, **(B)** is enlarged from a rectangle of **(A)**. Genes with upregulation are shown in red, and genes with downregulation are shown in green. Orange indicates predicted activated genes, blue indicates predicted inhibited genes, and an uncolored node indicates that the genes were not differentially expressed in this pathway. The IPAs were produced using QIAGEN's IPA (https://www.qiagenbioinformatics.com/products/ingenuity-pathway-analysis). The increase in expression over time in this canonical pathway, especially in the FcγR-associated pathways, was evident in the coculture model.

Signaling related to dendritic cell maturation, which is involved in the host innate immunity, was generally high in all models (Figure 2, Supplementary Figures 2, 3). Overall, the signaling of dendritic cell maturation was found to be higher with *z*-score values of 3.742, 2.714, and 4.564 in the 2-, 12-, and 24-h results in the coculture models. In the single cell line models, the *z*-score values were determined as high as 4.359, 4.041, and 4.333 at 2-, 12-, and 24-h results. In the coculture model, the *z*-score value shows a tendency to increase the expression with respect to the time, but in the single cell line culture model, the *z*-score value is maintained at a high value regardless of the time. The increase in expression with respect to the time in this canonical pathway, especially in the Fc gamma receptor- (FcγR-) associated pathways, was evident in the coculture model. The expression of DEGs associated with CD40, MYD88, Toll-like receptor (TLR), IL-1, TNF-α, and TNF-β associated with this pathway was found to be increased. In addition, TLR signaling, which is closely related to dendritic cell maturation and plays an important role in antigen presentation, has also been shown to increase expression at all-time points.

**Figure 3:**
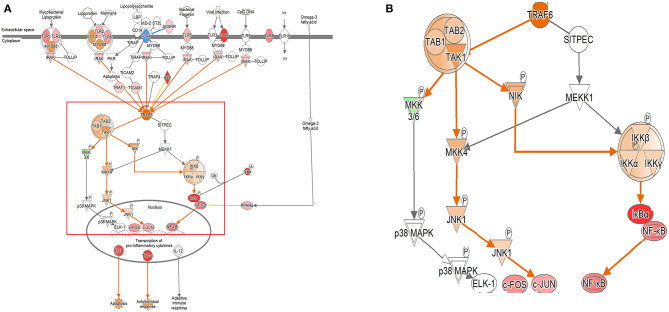
Ingenuity pathway analysis of the Toll-like receptor (TLR) signaling pathway in the DH82 cell line treated with *B. canis*. **(A)** TLR signaling pathway in 24 h on coculture model, **(B)** TRAF6-associated pathway on TLR signaling pathway, **(B)** is enlarged from a rectangle of **(A)** genes with upregulation are shown in red, and genes with downregulation are shown in green. Orange indicates the predicted activated genes, blue indicates the predicted inhibited genes, and an uncolored node indicates that the genes were not differentially expressed in this pathway. The IPAs were produced using QIAGEN's IPA (https://www.qiagenbioinformatics.com/products/ingenuity-pathway-analysis). A distinct change in gene expression was observed in the TRAP6-related pathway, and the coculture model confirmed that the expression of the related genes was increasing gradually.

In both the models, the expression of TLR signaling was observed to increase with time, and the expression was more pronounced in the single cell culture model (Figure 3, Supplementary Figures 4, 5). In the coculture model, the *z*-scores of 2, 12, and 24 h were measured as 1.663, 2.121, and 2.668 and gradually increased with time period. In the single cell line model, the *z*-scores of the 2-, 12-, and 24-h results were 2.333, 2.887, and 2.887, respectively, which were higher than those of the coculture model. A distinct change in the gene expression was observed in the TRAP6-related pathway, and the coculture model confirmed that the expression of the related genes was gradually increasing. On the other hand, in the single cell line culture model, it was found that the expression of the TRAF6-related pathway decreased in 24 h. However, the trend of increasing expression with time period was found to be similar. This pathway shows that the stimuli of the pathogen induce the expression of pro-inflammatory cytokines in the host. DEGs associated with TLR, MYD88, TRAF6, IL-1, TNF-α, and NF-κB pathwyas were identified. TREM1 signaling, which is related to the TLR and closely related to the host immune response to the pathogen, has also been shown to increase the expression.

**Figure 4:**
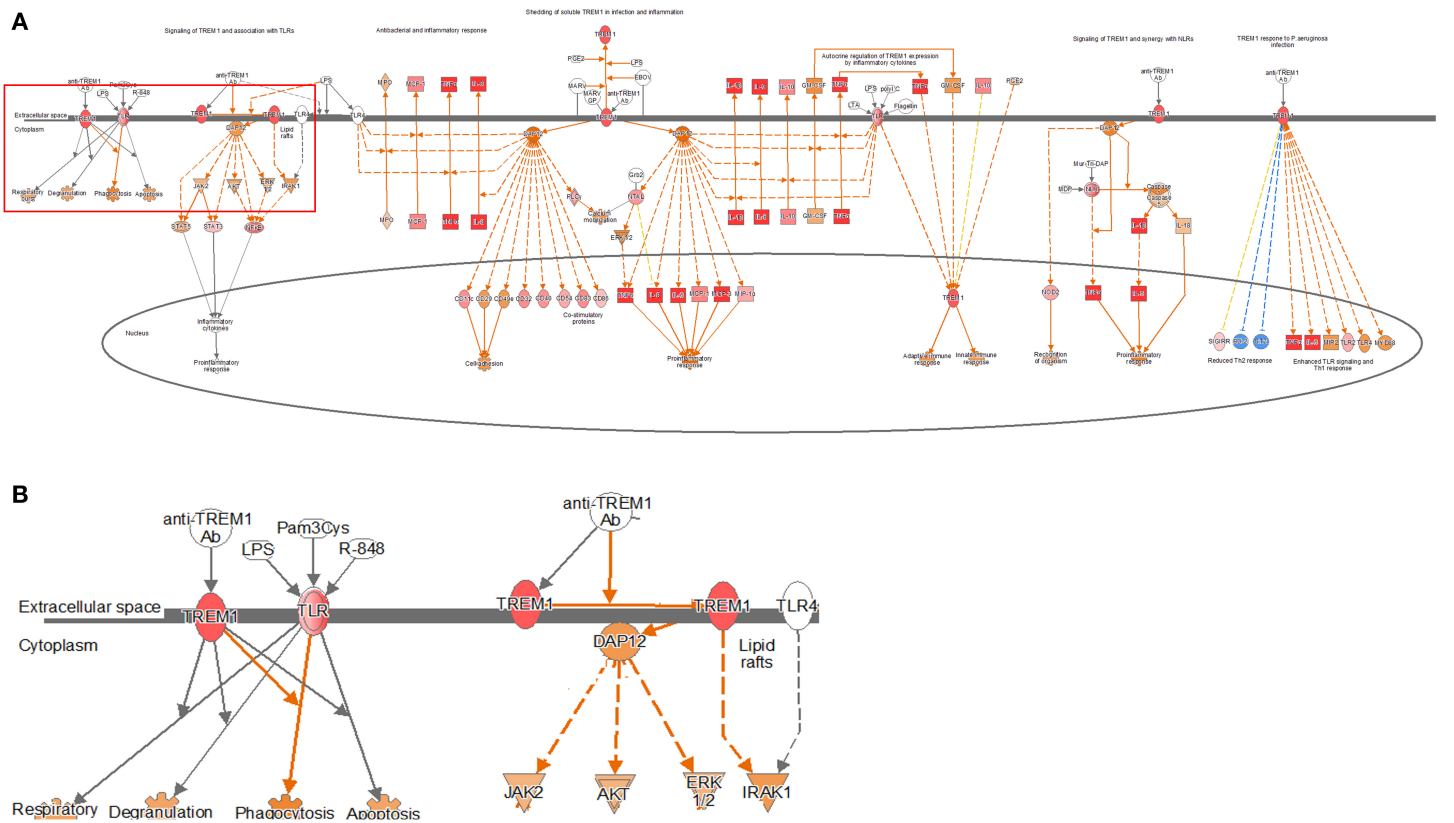
Ingenuity pathway analysis of the TREM1 signaling pathway in the DH82 cell line treated with *B. canis*. **(A)** TREM1 signaling pathway in 24 h on coculture model, **(B)** TREM1 and TLR-associated pathway on TREM1 signaling pathway, **(B)** is enlarged from rectangle of **(A)** genes with upregulation are shown in red, and genes with downregulation are shown in green. Orange indicates the predicted activated genes, blue indicates the predicted inhibited genes, and an uncolored node indicates that the genes were not differentially expressed in this pathway. The IPAs were produced using QIAGEN's IPA (https://www.qiagenbioinformatics.com/products/ingenuity-pathway-analysis). Both the models have been identified in this pathway, particularly on the pathways of TREM1 signaling with association to TLR, increasing over time.

TREM1 signaling, similar to the other pathways, was found to increase gradually over time in both the models, especially in the 24-h results of the coculture model (Figure 4, Supplementary Figures 6, 7). In the coculture model, the *z*-score values were 3.207, 3.606, and 5.112 at 2, 12, and 24 h, and increased with time. In addition, the *z*-score value was largely measured at the 24-h result. In the single-culture model, *z*-score values were measured as 3.771, 3.578, and 4.315 at 2, 12, and 24 h, respectively. Both the models have been identified in this pathway, particularly on the pathways of TREM1 signaling with an association to TLR, increasing over time. This result, similar to that observed with the coculture model, showed a tendency to increase over time, but was found to be a more gentle trend. The TREM1-associated pathway has also been activated to induce the expression of pro-inflammatory cytokines, similar to the TLR signaling pathway. An increased expression of DEGs, such as TREM1, TLR, IL-8, TNF-α, and DAP12 associated with this pathway was found.

**Figure 5:**
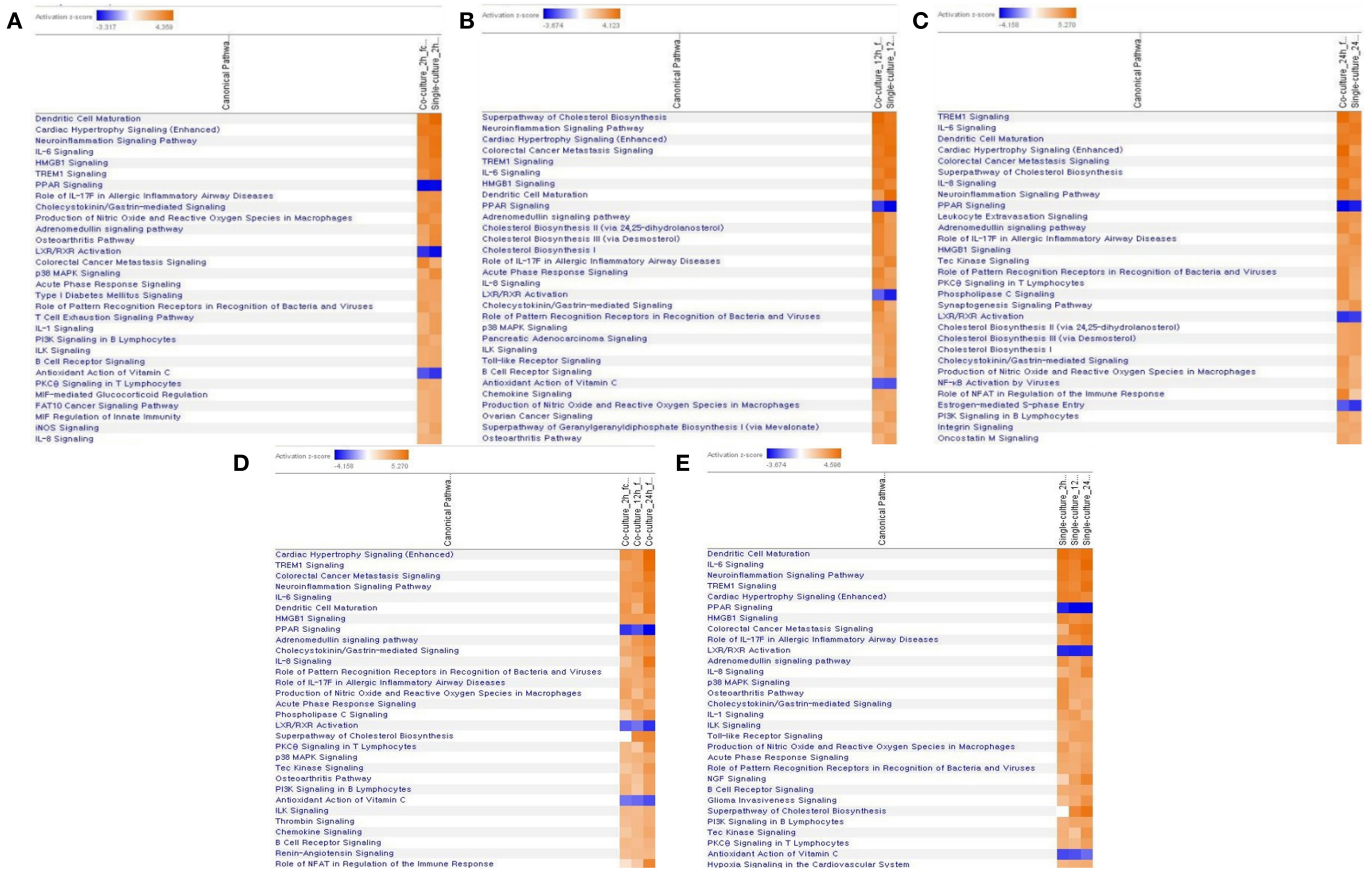
Comparative analysis of canonical pathway by each model and time period by *z*-score value. **(A)** In a 2-h top 30 canonical pathway comparison analysis of each model, **(B)** in a 12-h top 30 canonical pathway comparison analysis of each model, **(C)** in a 24-h top 30 canonical pathway comparison analysis of each model, **(D)** comparative analysis of the canonical pathway over time in a coculture model, **(E)** comparative analysis of the canonical pathway over time in a single-culture model.

In the comparison analysis, the 2-h results of both the culture models identified the pathways that are closely related to the cell immune responses, such as dendritic cell maturation, HMGB1 signaling, TREM1 signaling, IL-1 signaling, acute-phase response, the role of pattern recognition receptors (PRRs) in the recognition of bacteria and viruses, B-cell receptor signaling, and the IL-8 signaling pathway. Gene expression related to a communication between the innate and the adaptive immune cell was confirmed. At the 2- and 12-h time points of the early infection stage, the expression of acute-phase response signaling was high. The 12-h and the 24-h culture model also showed similar results to the 2-h culture model, but in the case of TNFR2 signaling, a differential regulation of the cytokine production in macrophage and T-helper cells by IL-17A and an acute-phase response, the 24-h model showed no high expression. In each model, the expression of canonical pathways with respect to time was commonly observed. In a single-culture model, the expression of genes associated with an acute-phase response was identified at all-time points. In the coculture model, the expression of genes was confirmed up to 12 h, but no expression was observed in the 24-h result. Most of the results showed the expression of the osteoarthritis pathway and neuroinflammation signaling pathway among the pathways that are not related to the cell immune response (Figure 5).

Changes in various DEGs related to dendritic cell maturation were identified. In FcγR, a difference in the expression was clearly observed according to the model. In the coculture model, the expression of FcγR was inhibited at the 2-h time point, but the expression was increased at 12- and 24-h time points. This finding is observed because the expression of two DEGs, except for FCGR1A, among FCGR1A, FCGR2B, and FCGR3A, which are related to FcγR and exhibit expression, is reduced (fold change value, FCGR1A: 1.240, FCGR2B:−2.099, and FCGR3A:−1.257). In contrast, in the single cell line culture model, the expression of FcγR increased at the 2-h result, but it was difficult to determine the expression pattern at 12- and 24-h results. This difficulty was due to the increase in FCGR1A at 2 h, but an increase in FCGR1A but a decrease in FCGR2B at 12 h (fold change value at 2 h, FCGR1A: 1.828, fold change values at 12 h, FCGR1A: 3.411, FCGR2B: −2.888). The 24-h results of the single cell line culture model were similar to the 12-h results, but the expression of FCGR1A increased but the expression of FCGR2B decreased (fold change value at 24 h, FCGR1A: 4.993, FCGR2B: −4.627).

Among the TREM1 signaling pathways, differences in pathways related to phagocytosis and apoptosis in the host were found. The 2-h results in the coculture model did not confirm the expression of TREM1- and TLR-related genes in relation to DEGs. However, at 12 h, the expression increased as the expression of TLR-related DEGs was confirmed. At 24 h, the expression of the pathway increased significantly, as the expression of not only TLR-related DEGs but also TREM1 increased. In the single cell culture model, the expression of TLR-related DEGs in the pathway associated with phagocytosis and apoptosis decreased by 2 h. However, as TLR-related DEGs increased at 12 and 24 h, the expression of related pathways also increased significantly.

In the case of TLR signaling, the differences were observed depending on the model. In the coculture model, the expression was reduced in the center of the TRAP6 gene for the 2- and 12-h results. However, the 24-h results showed an overall increase in the expression. When looking at the pattern over time, it was confirmed that the expression of TLR-associated DEGs gradually increased. The expression of TLR-related DEGs was not confirmed in the 2-h results, but the expression of TLR-related DEGs was confirmed in the subsequent results. The fold change values of TLR 1, 2, 3, 4, 6, 7, and 8 DEGs at 12 h were 1.602, 1.329, 3.531, 1.257, 1.474, 8.877, and 2.585, respectively. The expression of TLR 1, 2, 4, 5, 6, 7, and 8 DEGs was confirmed at 24 h, and the fold change values were 5.278, 2.990, 1.580, 2.751, 3.864, 42.224, and 5.426. All expression levels of TLR-related DEGs, which are commonly expressed, increased over time. In the single cell line culture model, TLR signaling was found to have an overall increase. When fold change values were confirmed at the genetic level, it was confirmed that the 2-h result was downregulated in some TLR-related DEGs (fold change values, TLR 1: −1.753, TLR 2: −1.301, TLR 4: 1.301, TLR 6: −2.395, and TLR 8: 1.347). The fold change values of DEGs increased over the 12-h period. The fold change value of TLR 2, TLR 7, and TLR 8 was 2.250, 22.785, and 3.272, respectively. The expression of more diverse TLR-related DEGs was observed to increase at 24 h. The expression of the TLR 1, 2, 4, 6, 7, and 8 DEGs was increased, and the fold change value of each was 4.141, 2.732, 1.223, 2.329, 29.651, and 4.563, respectively.

In addition, the expression of the “role of macrophages, fibroblast, and endothelial cells in rheumatoid arthritis,” “role of osteoblasts, osteoclasts, and chondrocytes in rheumatoid arthritis,” and the “neuroinflammation signaling pathway” associated with arthritis and neurological symptoms caused by the known *Brucella* species has been also observed (24, 30–33).

### Validation of RNA-Sequencing Results by Using Quantitative Real-Time PCR

**Figure 6:**
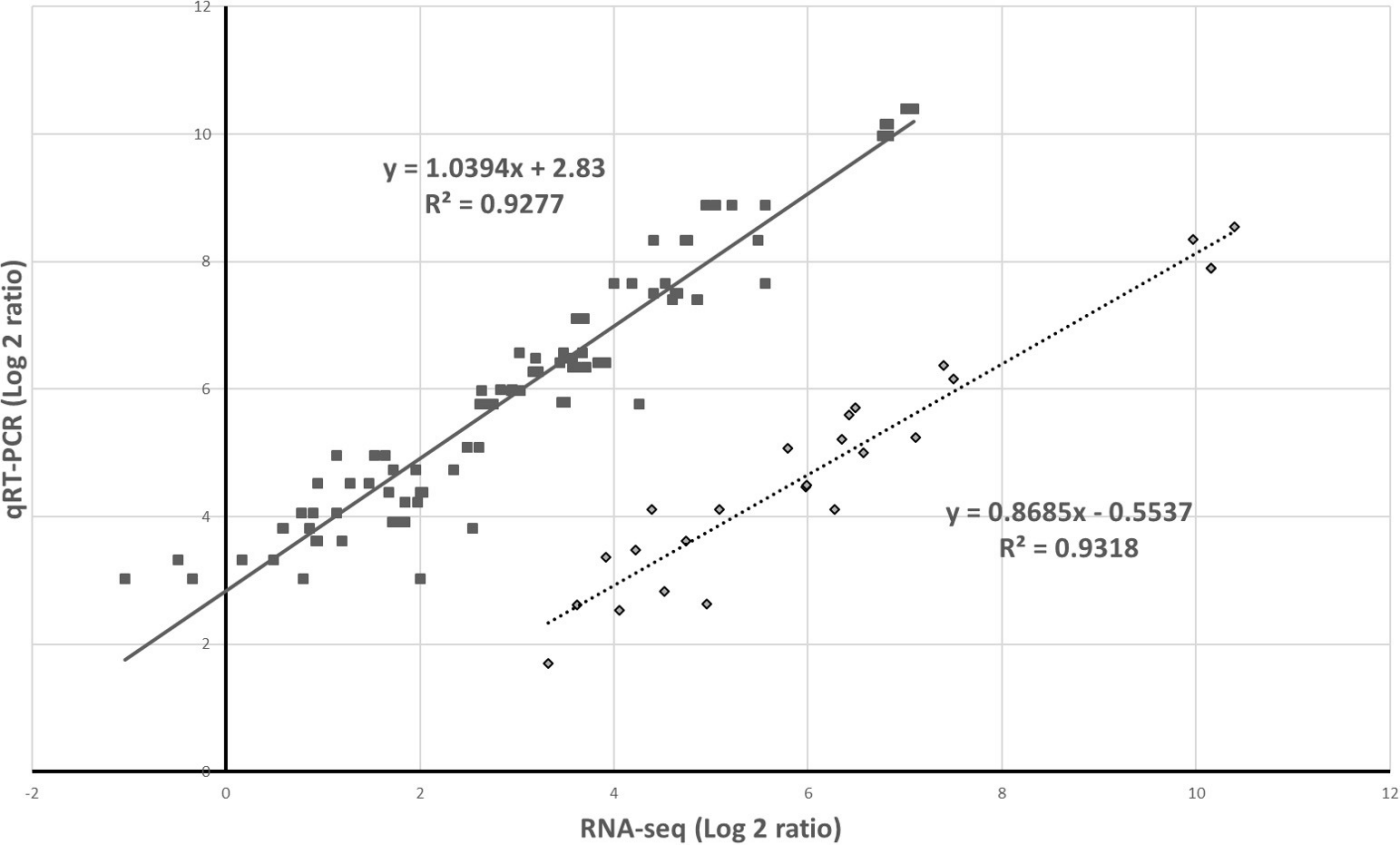
Validation of gene expression by RNA-sequencing (RNA-seq) and quantitative real-time PCR. The relative expression was compared to that observed in the controls to determine the fold change in the expression for each gene. This indicates the two independent experiments.

The RNA-seq results were verified following a qRT-PCR with samples from the two different experiments. The results of the five selected genes from the two analytic methods were highly correlated (Figure 6).

## Discussion

In this study, gene expressions were analyzed in the DH82 cell line treated with *B. canis* in the coculture model and the single cell line culture model. Most of the various signaling pathways identified as a result of DEGs were related to the host immune response. These pathways were commonly identified in the two models, and the degree of differences in the amount of expression in the related genes was identified. In particular, the expression of genes such as *CCL4, CCL5, CCL3L3, IL1A, IL1B*, and *SAA1* has been found to increase significantly in both the models in common. Genes such as *CCL4, CCL5*, and *CCL3L3* are all closely related to the activation of NF-κB, which is involved in the expression of cytokines related to the immune response. They are secreted by macrophages and play an important role in pro-inflammatory cytokines (34, 35). *SAA1* is a gene associated with serum amyloid A (SAA), an acute-phase protein, and is known to play a role in inflammatory reaction (36). This increase in the gene expression can be predicted to induce an early immune response from the host. Among the results, the expression of signaling pathways related to “adaptive immunity” was clearly observed. In particular, the signaling pathways related to IL-6, TLR, NF-κB, and TNFR2 signaling, which are related to dendritic cell maturation, were also markedly changed. The changes in DEGs related to immune response mechanisms associated with “cellular immune response” were also identified. In addition to HMGB1 signaling, these changes were also associated with the IL-6, NF-κB, IL-1α, IL-1β, and TNF-α signaling pathways. In addition, the expression of the innate immunity pathways that recognize and present antigens, such as pathogens, includes dendritic cell maturation, PRR signaling, and TLR signaling.

In the aforementioned pathways, the expression of pathways, such as “communication between the innate and the adaptive immune cell,” has been confirmed based on the same pathway, which suggests that the host immune mechanism is closely related to the innate immunity and the activity of the adaptive immunity following pathogen invasion. It can be observed that signaling related to acute-phase response is clearly expressed at 2 and 12 h in both the models by a comparison analysis. This finding suggests that *B. canis* usually develops chronically, but when the infection actually occurs, the host immune response is clear. The role of PRRs in the recognition of bacteria was also confirmed through the activity of TLR signaling in the cell membrane and NF-κB signaling, which induces pro-inflammatory cytokines.

In addition, this effect is known to be closely related to TREM1 signaling. TREM1 signaling is associated with pro-inflammatory cytokine activation. TREM1 activation triggers signals such as JAK2 and STAT3 and affects signals, such as NF-κB. TREM1 signaling is closely related to TLR signaling, and the synergy of the two produces neutrophil degranulation, phagocytosis, and the respiratory burst but also produces pro-inflammatory cytokines (37–39).

Dendritic cells are among the most efficient antigen-presenting cells (APCs) of the immune response system. Immature dendritic cells are responsible for capturing and processing antigens and for presenting major histocompatibility complex (MHC)-specific antigens in secondary lymphoid organs. After antigen capture, dendritic cells mature, antigen capture capacity is downregulated, and costimulatory molecules and MHC class I and II molecules are upregulated to enhance the antigen presentation. Matured dendritic cells have the ability to produce the cytokines that can enhance the innate and the adaptive immune response and have the ability to cross exogenous antigens to cytotoxic lymphocytes (40). In most APCs, peptides derived from exogenous antigens introduced from the extracellular environment are preferentially present in CD4+ T cells in MHC class II molecules. However, the antigens that are internalized in dendritic cells are also present in CD8+ T cells through a cross-presentation (41). The interaction between the peptide-loaded MHC class I molecules and the T-cell receptor alone is not sufficient to initiate the T-cell response. Therefore, an induction of CD8+ T-cell responses *in vivo* by antigens internalized by dendritic cells is achieved. Additional signaling of costimulatory molecules and cytokines is also essential for the development of effective T-cell responses. The pathogen is detected through a pattern of recognition receptors such as TLRs, present on dendritic cells, and activated TLRs trigger the mitogen-activated protein kinase (MAPK) pathway to induce the activation of transcription factors such as NF-κB (42, 43). This effect increases the expression of costimulatory molecules, including CD40 and CD80, and promotes the release of various inflammatory cytokines and chemokines (44). The FcγR of dendritic cell maturation pathway is closely related to the phagocytosis of microbes and is associated with a combination of the IgG molecule.

In many situations, dendritic cells are simultaneously stimulated by antigens and danger signals. This stimulation occurs, the release of the TLR ligand occurs and the innate sensor is associated with the endosomes and phagocytosis. In fact, the expression of various TLR DEGs was increased in the dendritic cell maturation signaling pathway identified in this study, and the expression of CD40 and MAPK pathways was increased. Changes in various DEGs related to dendritic cell maturation were identified. The expression of DEGs for related cytokines, such as IL-1, IL-6, IL-10, and IL-12, was found to be increasing in all the results, which induced dendritic cell maturation. FcγR is commonly known to be associated with the activation of dendritic cells, and this study has identified the expression of MHC I, II, and CD40 with the actual expression of FcγR (45). This result may be evident that dendritic cell maturation plays an important role in early *B. canis* infection and induces the host immune response. However, previous reports on *Brucella abortus* or *B. melitensis* suggested the suppression of host dendritic cell maturation signaling by the stimulation of *Brucella* spp. (46, 47). Those disagree with our results showing the activation of the dendritic cell maturation signaling. The disagreement might be due to a difference in structural components of the bacterium, which is used in each experiment. Opposite direction of the expression of the signaling could be related to the different chemical inducers of cell components because they used a smooth form of *Brucella* spp. (46, 47). However, the reality of the difference should be revealed in future studies.

TREM1, a triggering receptor expressed on myeloid cell 1, is an activation receptor expressed on myeloid cells included in the Ig superfamily (48). TREM1 plays an important regulatory role in the innate immune response, and early research has focused primarily on the role of TREM1 in lipopolysaccharide-induced sepsis (49). This gene is known to be induced at high levels in neutrophils, monocytes, and macrophages, which intensify the secretion of pro-inflammatory cytokines and chemokines in response to bacterial infections, further amplifying the TLR initiation response to microorganisms (49, 50). The effects of TREM1 signal transduction on microbial control are controversial. Infections with *Leishmania major* or influenza virus did not affect removal but were known to have been effective in *Klebsiella pneumonia* and *Pseudomonas aeruginosa* clearance (51, 52). Especially in the case of infection with *P. aeruginosa*, TREM1 contributed to this through mechanisms involved in the migration of neutrophils for the removal of pathogens (53). In addition, animal experiments confirmed that the mortality of Gram-positive or Gram-negative bacteria-infected TREM-1/3-deficient mice was significantly increased compared to wild-type mice (53, 54). In the TREM1 signaling pathway, DAP12 plays an important role. DAP12 is responsible for inducing intracellular signaling within the TREM1 signaling pathway, leading to the production of chemokines and cytokines through the phosphorylation of DAP12 (55). In this study, the expression of TLR as well as DAP12 was confirmed with the expression of TREM1, and the associated genes were also identified, resulting in the activation of TREM1 signaling pathway. This finding shows that TREM1 signaling plays an important role in the innate immune response. However, to date, the study of TREM1 has not thoroughly elucidated whether this protein participates in and regulates innate immune responses against other pathogenic microbial groups. In this study, the expression of TREM1 signaling exhibited high *z*-scores in all the models, and it was confirmed that the expression showed an overall increase with respect to time. The results of this study confirmed that the dendritic cell maturation and TLR signaling pathways were activated and TREM1 signaling was also activated. This finding may be the reason why there is a close association between the activation of TLR and TREM1 signaling. The expression of the IL-6 and IL-8 signaling pathways occurred, which may be closely related to the activation of TREM1 signaling.

The TLR family is a part of the widely studied PRR class. TLRs have been identified in 10 humans and 12 murines, which play a role in recognizing intracellular and extracellular pathogen-associated molecular patterns (PAMPs). TLRs 1, 2, 4, 5, 6, and 11 are expressed in cell membranes, and TLRs 3, 7, 8, and 9 are present in endosomes in cells (43, 56). These receptors are found in a broad range of cell types, such as dendritic cells, macrophages, NK cells, B cells, and T cells. TLR2 usually recognizes a wide range of microbial molecules, such as peptidoglycans, lipoproteins, and yeast polysaccharides (57). TLR4 recognizes LPS and several viral envelope proteins, and TLR5 plays a role in recognizing flagella, the pathogenic factor of motility bacteria (58, 59). TLR3, -7, -8, and -9 recognize nucleic acids produced in viruses and bacteria, and all TLRs achieve the recognition of endogenous ligands according to inflammatory and autoimmune diseases (43). The role of TLRs in *Brucella* infection has been studied in mouse models using *B. abortus, B. melitensis, Brucella ovis, and Brucella microti*, and is known to play a key role in immune function (60–63). TLR signaling is closely related to dendritic cell maturation and TREM1 signaling pathways.

The pathways characterized by a manifestation in major canonical pathways are mostly associated with pro-inflammatory cytokines, which are known to be associated with the early immune response of a host. These results show that the infection of *B. canis* induces the early immune response from a host and is linked to phagocytosis.

As dendritic cell maturation is performed, the TLR signaling expression is increased to play an antigen-presenting role. In this study, the expression of TLR signaling gradually increased as dendritic cell maturation increased in both models. In particular, TLR7 was found to change the fold change value up to 40 times, unlike other TLR-related DEGs. Recent studies on TLR3 and TLR7 have shown that they are involved in the detection of *Brucella* RNA by the host. The production of cytokines, such as IL-6, IL-12, and TNF-α, induced by *B. abortus* RNA is known to be TLR-dependent and occurs through the signaling of MAPK and NK-κB signaling (64). Furthermore, a previous study showed the increase in TLR2 and TNF-α as the host immune responses to the infection of *B. abortus* through the host immune response to pathogen infection (65). When infected with the other *Brucella* spp. infections, TLR2, 4, 5, and 9 were mainly expressed, and these were the main concerns (60, 61). TLR7 expression was significantly increased in our study with *B. canis*. Usually, TRAF6 is identified primarily in the signaling pathway *via* CD40, but it has been confirmed that the TLR stimulates the activation of the TRAF6 pathway, and in this study, the activation of the TRAF6 pathway also confirms the activation of IKK and NF-κB (66). The increase in the expression of TLR7 led to an increase in the expression of TRAF6, which in turn induced the expression of NF-κB. This translates the expression of pro-inflammatory cytokines. Our results suggested the importance of the role of TLR7 in the early stage of infection of *B. canis*. However, the role of TLR7 in the infection of *Brucella* spp. seem to be not completely recognized, yet even though the results of this study suggest that TLR7 plays an important role in the infection of *B. canis*.

As is well-known, *B. canis* infection is likely to be closely associated with TLR as the other *Brucella* species. In addition, the expression of pathways, such as dendritic cell maturation, acute-phase response signaling, and TREM1 signaling, associated with early immunity suggests that *B. canis* has been active in the host since early infection. Through these pathways, the expression of pro-inflammatory cytokines, such as TNF-α, IL-1, and IL-6 signaling, was confirmed. In this study, high expression patterns of the pathways that interact with innate immunity, such as dendritic cell maturation, TREM1, and TLR signaling, were identified. Dendritic cell maturation and TLR signaling provide an immune response to pathogen invasion into the host, as well as continuous antigen recognition, while dendritic cell maturation and TREM signaling complement each other to induce a more effective immune response. These effects are well-known to recognize pathogens in the host and play a very important role in the initial immune response.

Based on the RNA-seq analysis, the host immune response to the infection of *B. canis* was analyzed at the transcriptomic level. No significant differences were identified in the pathways expressed between the two models, but the differences were identified in the degree of genes expressed in the pathways. These results suggest that, although *Brucella* infections generally occur chronically, they respond to active host immune activity in the early stage of infection. This effect may be an important indicator of *B. canis* infection. Dendritic cell maturation and TREM1 signaling are all closely related to the TLR signaling, all of which are closely related to the host early immune response. In addition, dendritic cell maturation confirmed that the communication between the innate and the adaptive immunity to the infection is not only the innate immunity of the host immune response but also the promotion of the adaptive immunity and the achievement of the immune response.

By using the coculture model, a model similar to the *in vivo* environment through the interaction between the epithelial cells and the macrophage was established. Gene expressions at the transcriptomic level were similarly identified in the two models, but a difference was found in the amount of expression. Despite the same proportion of pathogens, this difference in expression level has been identified, possibly where a preemptive defense function may have been activated as the pathogen undergo an epithelial cell. It is thought to be the host defense system that minimizes the infection damage through the primary protection against the pathogen infection in epithelial cells. However, further research on the role of epithelial cells in the infection should be considered to reveal the underlining mechanism of these models.

The results of this study have been analyzed at the genetic level of the host immune response, free from the investigation of the host simple immune response, isolation, and epidemiology. High expression of pathways, such as dendritic cell maturation, TREM1 signaling, and TLR signaling, provided more specific evidence of the host early immune response to *B. canis* infection, and the changes in the gene expression contained in these pathways may be useful references for the early diagnosis of *B. canis*.

## Data Availability Statement

The datasets presented in this study can be found in online repositories. The names of the repository/repositories and accession number(s) can be found at: https://www.ncbi.nlm.nih.gov/geo/query/acc.cgi?acc=GSE134331, GSE134331.

## Ethics Statement

All experiments were reviewed and approved by the Seoul National University Institutional Biosafety Committee (protocol: SNUIBC-R180912-3).

## Author Contributions

WP conceived and designed the experiments, data curation, formal analysis, software, and writing the original draft. SK data curation, formal analysis, and software. SS data curation and formal analysis. HY conceptualization, project administration, supervision, and writing—review and editing. All authors contributed to the article and approved the submitted version.

## Conflict of Interest

The authors declare that the research was conducted in the absence of any commercial or financial relationships that could be construed as a potential conflict of interest.
