# Supplementary material for: Identification of Dendritic Cell Maturation, TLR, and TREM1 Signaling Pathways in the Brucella canis Infected Canine Macrophage Cells, DH82, Through Transcriptomic Analysis
Source: Front Vet Sci. 2021 Mar 19;8:619759. doi: 10.3389/fvets.2021.619759 (PMC8020338; doi:10.3389/fvets.2021.619759)
Supplement: Supplementary file 1 [file Data_Sheet_1.PDF]

Supplementary Material

1 Supplementary Figures

(A)

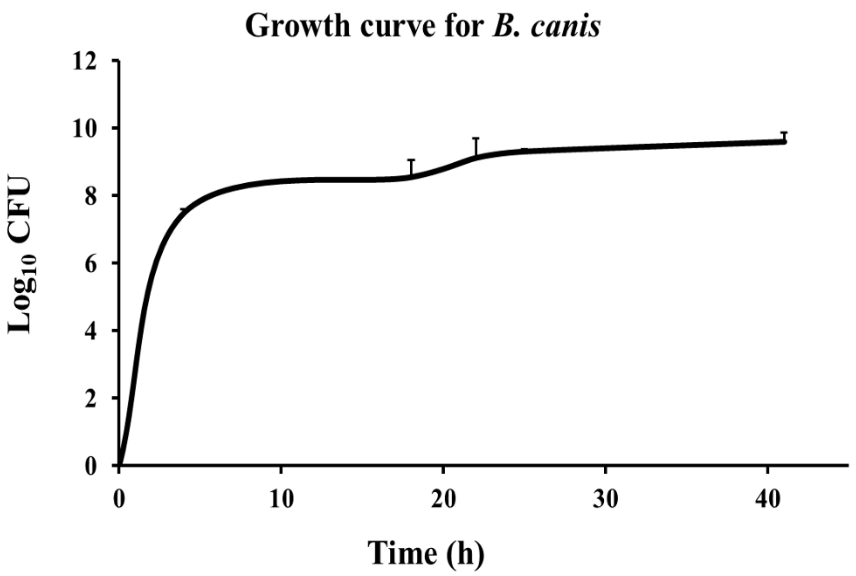

(B)

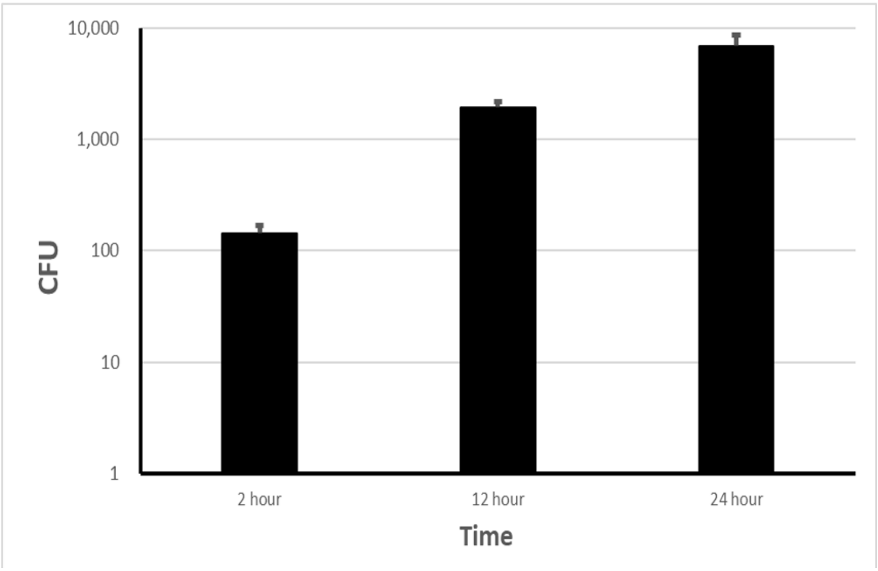

Supplementary Figure 1. (A) Growth curve over time of *B. canis* (B) Colony forming units of *B. canis* in the single cell culture model using DH82 cell line at 2, 12, and 24 hours after removal of antibiotics treatment in the invasion assay.

(A)

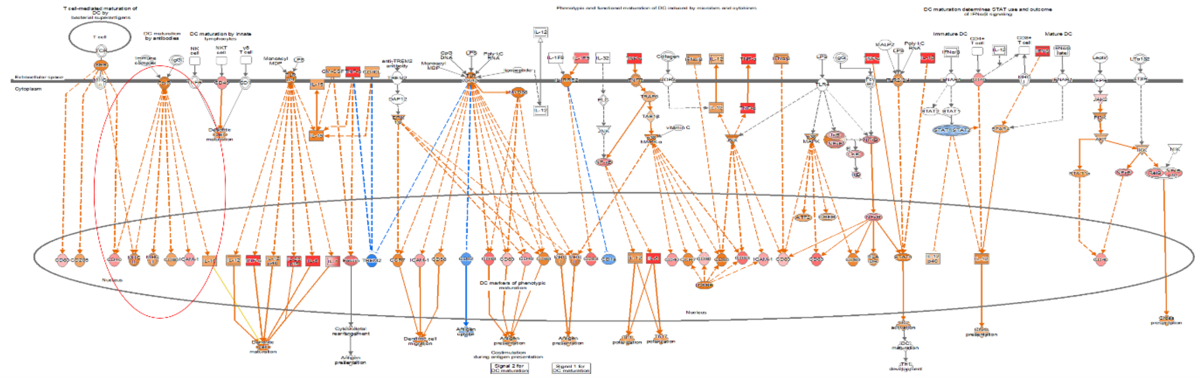

(B)

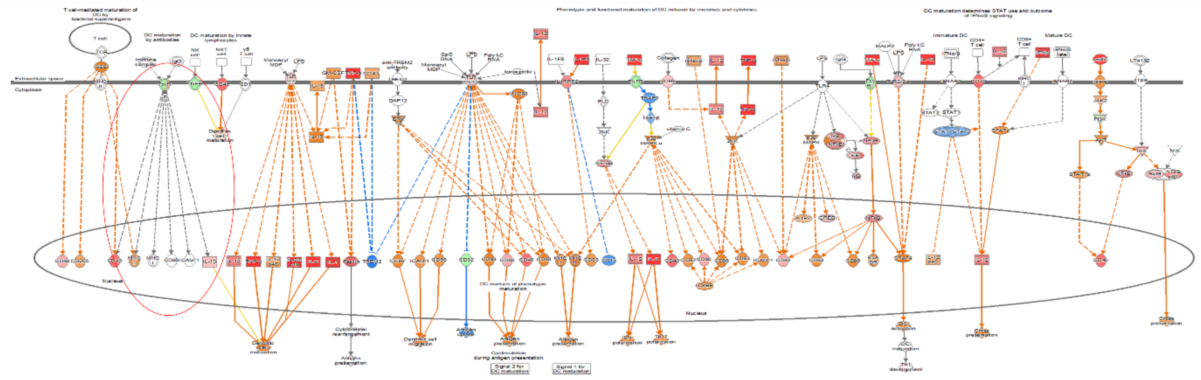

(C)

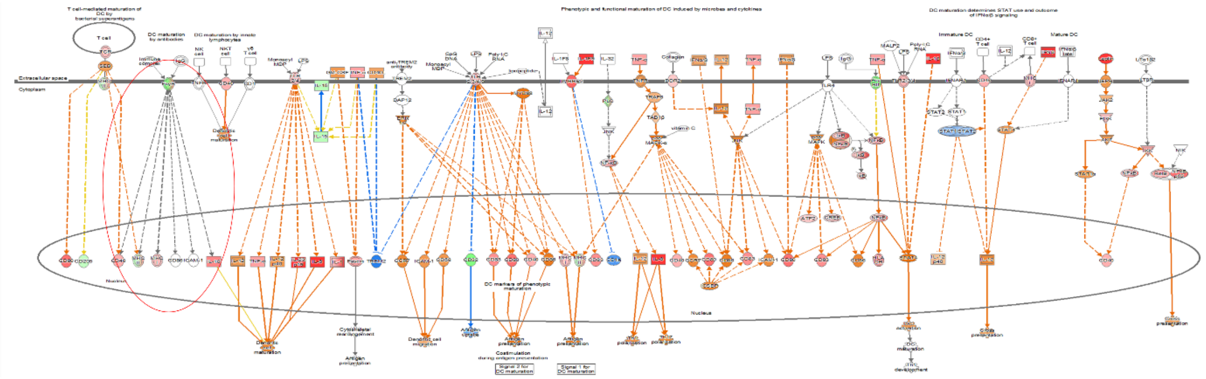

Supplementary Figure 2. Ingenuity pathway analysis of the dendritic cell maturation signaling pathway in the DH82 cell line treated with *B. canis* in the co-culture model. Pathways indicated at the different time points (A) at 2 hours, (B) at 12 hours and (C) at 24 hours. Genes with upregulation are shown in red, and genes with downregulation are shown in green. Orange indicates predicted activated genes, blue indicates predicted inhibited genes, and an uncolored node indicates that the genes were not differentially expressed in this pathway. The Ingenuity pathway analyses were product through the use of IPA (QIAGEN Inc, https://www.qiagenbioinformatics.com/products/ingenuity-pathway-analysis).

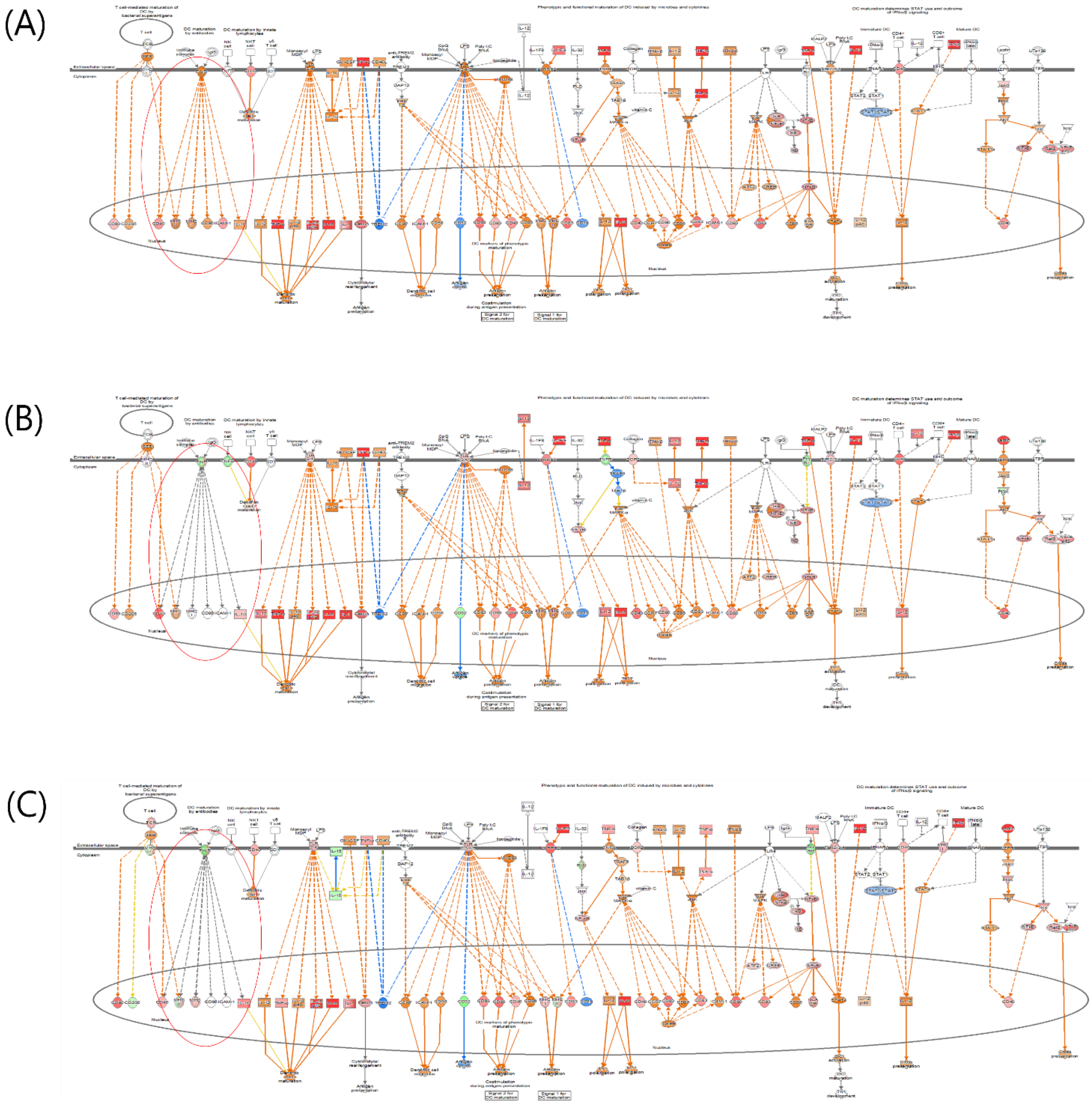

Supplementary Figure 3. Ingenuity pathway analysis of the dendritic cell maturation signaling pathway in the DH82 cell line treated with *B. canis* in the single cell line culture model. Pathways indicated at the different time points (A) at 2 hours, (B) at 12 hours and (C) at 24 hours. Genes with upregulation are shown in red, and genes with downregulation are shown in green. Orange indicates predicted activated genes, blue indicates predicted inhibited genes, and an uncolored node indicates that the genes were not differentially expressed in this pathway. The Ingenuity pathway analyses were product through the use of IPA (QIAGEN Inc, https://www. qiagenbioinformatics.com/products/ingenuity-pathway-analysis).

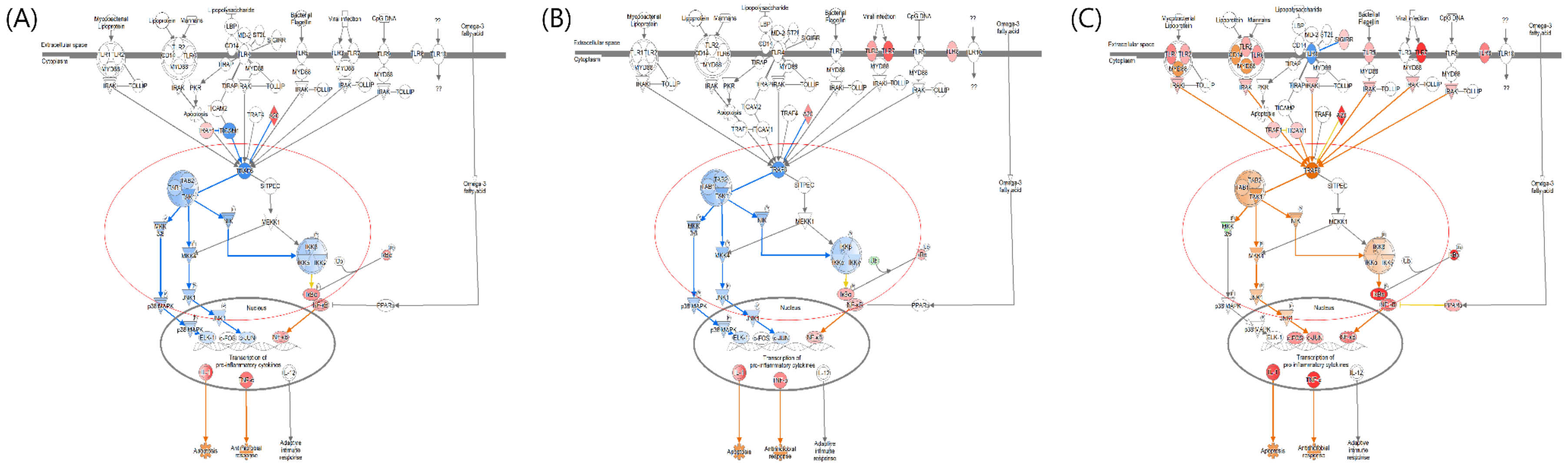

Supplementary Figure 4. Ingenuity pathway analysis of the Toll-like receptor signaling pathway in the DH82 cell line treated with *B. canis* in the co-culture model. Pathways indicated at the different time points (A) at 2 hours, (B) at 12 hours and (C) at 24 hours. Genes with upregulation are shown in red, and genes with downregulation are shown in green. Orange indicates predicted activated genes, blue indicates predicted inhibited genes, and an uncolored node indicates that the genes were not differentially expressed in this pathway. The Ingenuity pathway analyses were product through the use of IPA (QIAGEN Inc, https://www. qiagenbioinformatics.com/products/ingenuity-pathway-analysis).

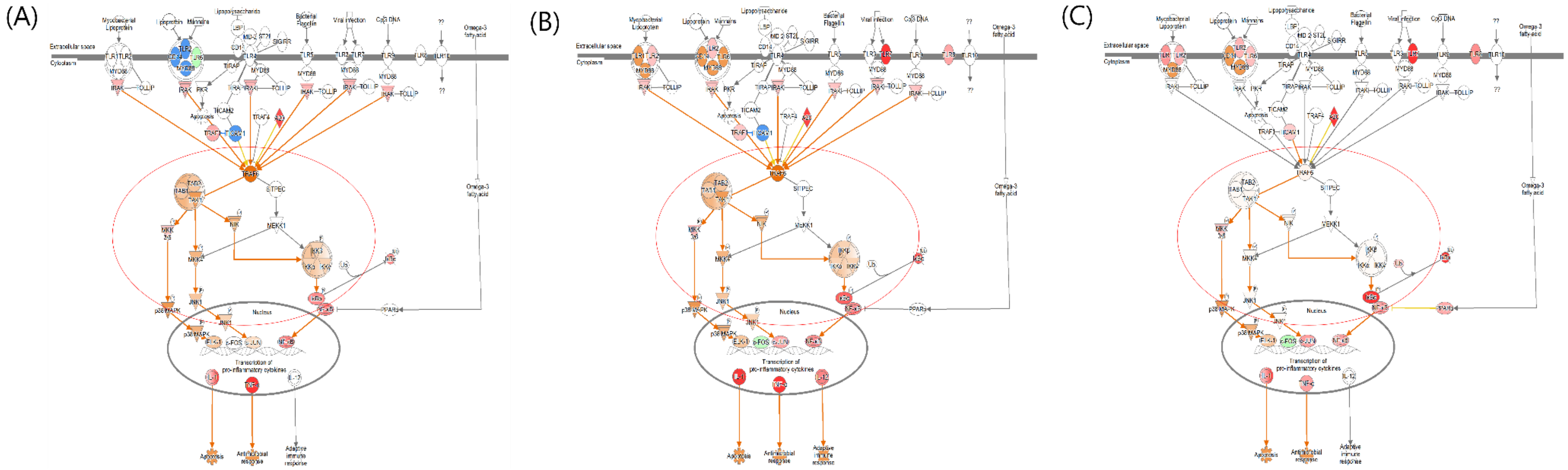

Supplementary Figure 5. Ingenuity pathway analysis of the Toll-like receptor signaling pathway in the DH82 cell line treated with *B. canis* in the single cell line culture model. Pathways indicated at the different time points (A) at 2 hours, (B) at 12 hours and (C) at 24 hours. Genes with upregulation are shown in red, and genes with downregulation are shown in green. Orange indicates predicted activated genes, blue indicates predicted inhibited genes, and an uncolored node indicates that the genes were not differentially expressed in this pathway. The Ingenuity pathway analyses were product through the use of IPA (QIAGEN Inc, https://www. qiagenbioinformatics.com/products/ingenuity-pathway-analysis).

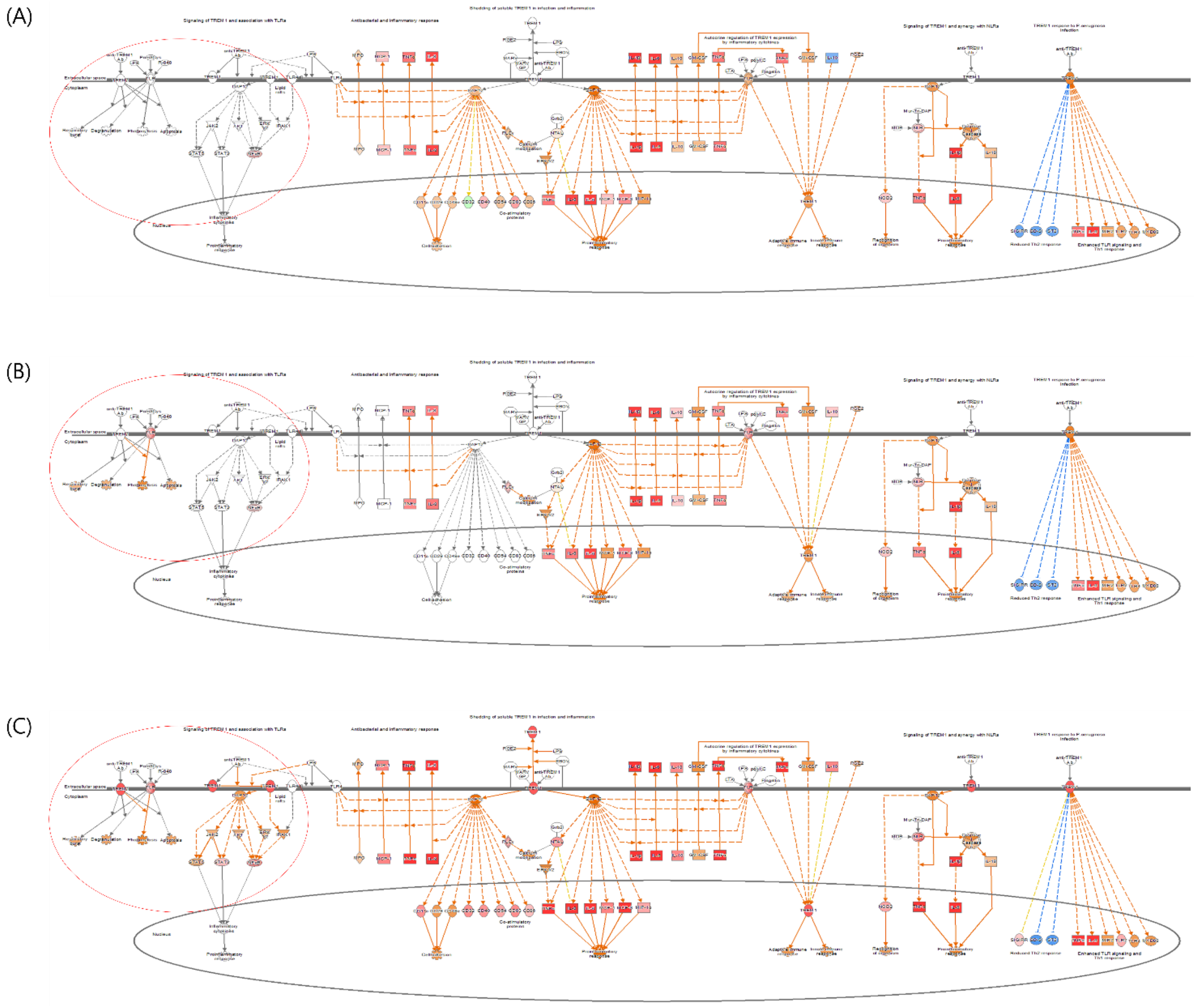

Supplementary Figure 6. Ingenuity pathway analysis of the TREM1 signaling pathway in the DH82 cell line treated with *B. canis* in the co-culture model. Pathways indicated at the different time points (A) at 2 hours, (B) at 12 hours and (C) at 24 hours. Genes with upregulation are shown in red, and genes with downregulation are shown in green. Orange indicates predicted activated genes, blue indicates predicted inhibited genes, and an uncolored node indicates that the genes were not differentially expressed in this pathway. The Ingenuity pathway analyses were product through the use of IPA (QIAGEN Inc, https://www. qiagenbioinformatics.com/products/ingenuity-pathway-analysis).

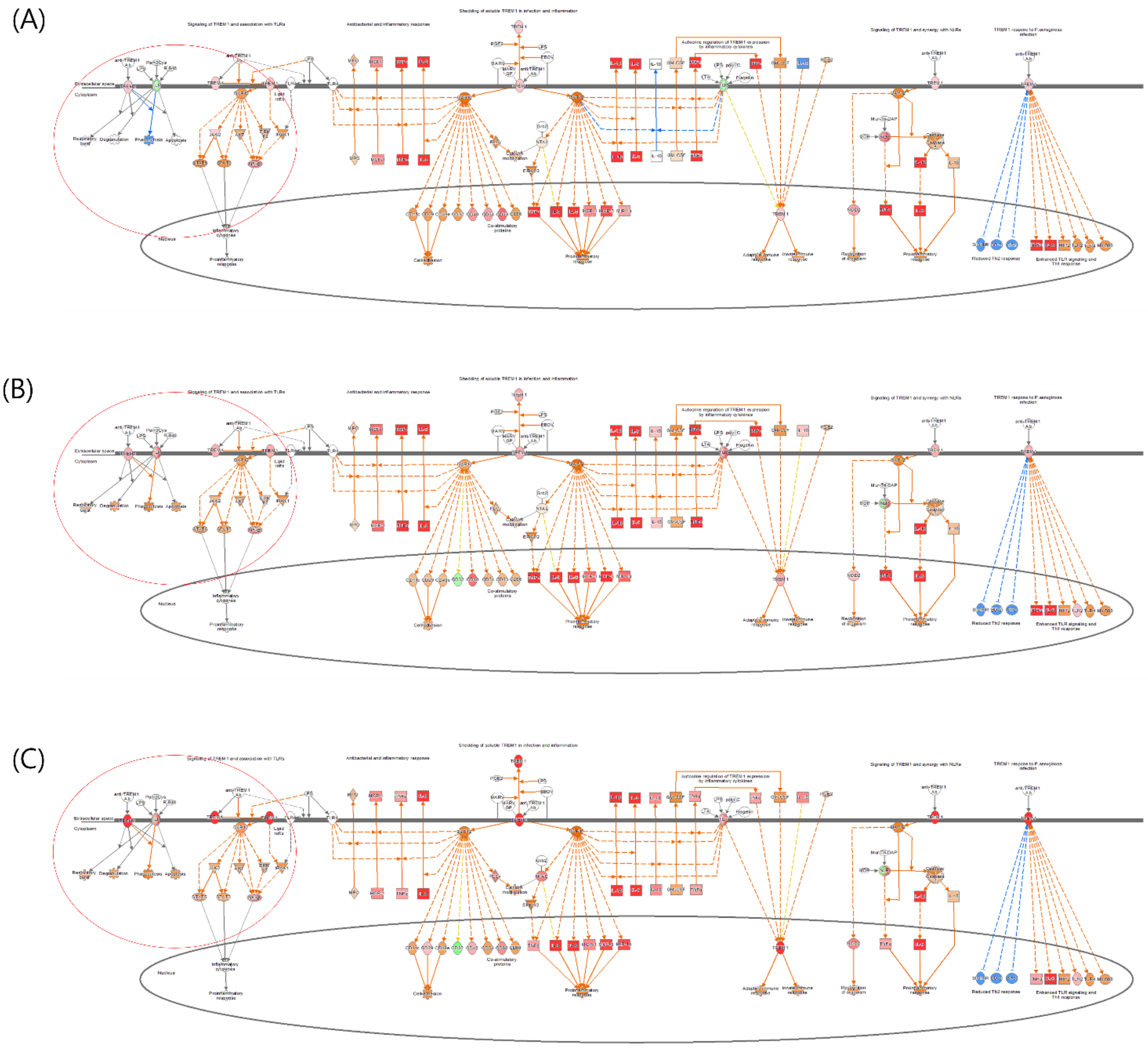

Supplementary Figure 7. Ingenuity pathway analysis of the TREM1 signaling pathway in the DH82 cell line treated with *B. canis* in the single cell line culture model. Pathways indicated at the different time points (A) at 2 hours, (B) at 12 hours and (C) at 24 hours. Genes with upregulation are shown in red, and genes with downregulation are shown in green. Orange indicates predicted activated genes, blue indicates predicted inhibited genes, and an uncolored node indicates that the genes were not differentially expressed in this pathway. The Ingenuity pathway analyses were product through the use of IPA (QIAGEN Inc, https://www. qiagenbioinformatics.com/products/ingenuity-pathway-analysis).
